# Development of spray-dried powder hand sanitiser with prolonged effectivity

**DOI:** 10.1038/s41598-024-55503-w

**Published:** 2024-02-28

**Authors:** Lucie Večerková, Lucie Mašková, Zdeněk Knejzlík, Ondřej Kašpar, Viola Tokárová

**Affiliations:** 1 Department of Chemical Engineering, University of Chemistry and Technology Prague, Technická 5, 166 28 Prague 6, Czech Republic; 2 Institute of Organic Chemistry and Biochemistry, Czech Academy of Sciences, Flemingovo náměstí 542/2, 160 00 Prague 6, Czech Republic

**Keywords:** Chemical engineering, Drug delivery, Antiparasitic agents, Disease prevention, Health care, Epidemiology

## Abstract

Since the outbreak of the COVID-19 pandemic, the use of hand sanitisers has become an inseparable part of our personal hygiene. However, the short-term effect and the need for frequent application are shortcomings that impair the overall protection. Another aspect is that repeated use of some products (typically alcohol-based) may cause skin irritation or eventually more severe health problems. This work proposes spray-drying as a suitable method for the preparation of swellable chitosan carriers, allowing for encapsulation and sustained release of antibacterial chlorhexidine digluconate as a model active substance. After application to hands, micron-sized particles preferentially accommodate space between epidermal ridges, protected against attrition. Thanks to their small size (d < 10 µm), particles are comfortable to carry since they are not recognisable by somatosensory receptors. The performance of formulations with various amounts of chlorhexidine and cross-linker was tested and compared with selected commercial disinfectants available on the Czech market (ethanol gel and alcoholic solution with chlorhexidine) against *E.* *coli* and *S.* *epidermidis*. The real-life performance was investigated with twelve volunteers performing various activities for up to 2 h. Finally, a replica of the human index finger with accurately captured micro-topology was proposed and compared with volunteers’ fingers concerning the total amount of adhered and detached particles.

## Introduction

Keeping hands clean is one of the fundamental habits that humanity has adopted as an integral part of its culture or religion long before the discovery of microorganisms and viruses. Despite this, the physical transmission of infection from person to person represents a serious problem that leads to an excessive burden on the health system, considerable economic impact on society, and a high number of deaths not limited to developing countries^1^.

It is wishful thinking that the grim picture of faces obscured by protective face masks is over for good. It was not so long ago when the COVID-19 pandemic changed overnight numerous aspects of our everyday life we have considered guaranteed. Increasing awareness of the global population and escalating demand for hand sanitisation products during the pandemic resulted in temporary supply shortages, increasing raw materials prices, and a search for homemade alternatives to traditional handwashing in regions with limited water access^2^. In the following years, we have accepted the omnipresence of hand-sanitising stations scattered around heavily populated urban areas. As a result, hand sanitisers in multiple forms, e.g., foam, gel, cream, wipe, or spray, have become an inseparable part of our hygiene habits, preventing the direct transmission of pathogens from a contaminated surface or person to person^3^. Pocket hand sanitisers have quickly found their place in our everyday lives, so we are more than ever aware of their practical benefits and limitations.

Hand sanitisers can be categorised into two groups: alcohol-based and alcohol-free. Alcohol-based sanitisers (60–95% v/v alcohol—ethanol, isopropanol, n-propanol) are the most frequently used commercial sanitisers. They are considered the gold standard by the World Health Organization (WHO)^1^ since they can dissolve the lipid membranes, denature bacterial proteins and inactivate viruses in 15–30 s^4,5^. However, these products provide only short-term protection, given the fast evaporation rate^6^. Also, the sanitiser efficacy is highly compromised when applied to dirty or greasy hands. For long-term protection, such sanitisers must be applied frequently, which is reflected in the final cost and overall efficacy, heavily relying on user compliance. Moreover, frequent use of alcohol products, e.g., by medical personnel, can lead to skin dryness by removing sebum, irritation, or severe health problems, i.e., contact dermatitis^7,8^. It should also be mentioned that alcohol use is strictly prohibited for various religious and cultural aspects, e.g., Buddhism, Sikhism, Hinduism, and Islam^9^. However, alcohol use as a medical agent for hand hygiene and medicinal purposes is generally tolerated if it cannot be substituted.

Alcohol-free products rely on various antiseptic agents with unique inhibition mechanisms on living tissues. Many of these agents belong to over-the-counter drugs available without a prescription. The commonly used antiseptics in hand sanitisers are hydrogen peroxide, chloroxylenol, iodine and iodophors, triclosan, benzalkonium chloride, and chlorhexidine^3^. The last mentioned was used as the model antiseptic agent in this work.

Chlorhexidine digluconate (CHG), a symmetric bis-biguanide with two positive charges at physiological pH^10^, was synthesised in 1954 during screening for active agents against malaria by Imperial Chemical Industries (Manchester, United Kingdom)^11^. CHG is a broad-spectrum biocide effective against gram-positive and gram-negative bacteria, enveloped viruses, and fungi. CHG binds to negatively charged microbial surfaces, interferes with the membrane transport system, and causes cytoplasmatic proteins and nucleic acids precipitation, followed by cell apoptosis^12,13^. Regarding its topical use, it is improbable for CHG to be significantly absorbed into the lipid-rich environment of the skin due to its chemical properties. CHG, a potent base with a p*K*a value of 10.8, mostly exists in the cationic form in the pH range of human skin (4.1–5.8)^14^. Poor penetration of CHG into the deeper skin layers was demonstrated in 24-h skin permeation studies using Franz diffusion cells^15^.

Controlled release of active ingredients through particles and carriers, triggered by stimuli like temperature, pH, magnetic and electric fields, is gaining popularity in pharmaceuticals^16,17^, cosmetics (skincare products^18^), and agro-industries (smart fertilisers^19^). Encapsulating antimicrobial agents, such as synthetic and natural antibiotics^20^, enzymes or inorganic nanoparticles^21^, play an important role in treating or preventing infections. For instance, CHG encapsulated in silica nanoparticles has been previously tested on skin to heal chronic wounds^22^. Chitosan/aloe film with sustain-released and delivery of curcumin shows an effect on restraining inflammation and cicatrices and promotes the intrinsic regeneration of skin tissues^23^. Another study proposes the use of antimicrobial thin and porous copper nanomesh acting as a wearable protective second skin^24^.

To provide comfort for the end user and, at the same time, increase the overall reliability of hand sanitiser, the effect of biocidal preparations needs to be prolonged from minutes to a few hours. This study presents swellable chitosan microparticles with incorporated CHG as a model antiseptic substance prepared by spray drying. The influence of cross-linker amount (TPP) and CHG content on particle size, shape and surface morphology was described along with the release kinetics of CHG. The size of CHG carriers was tailored such that particles, otherwise non-detectable by touch, occupy the void space between papillary ridges, thereby being physically protected from mechanical attrition, i.e., hand rubbing or touching. This experimental study confirmed that CHG physically encapsulated in swellable chitosan carriers could be gradually liberated into the surroundings, leaving hands protected against transferred pathogens for a prolonged time. The study involved testing antiseptic particles on volunteers to confirm the presence of particles and antimicrobial activity even after 2 h of intensive activity. The results were compared to two commercial hand sanitisers.

## Materials and methods

### Materials

Chitosan—low molecular weight, phosphate-buffered saline tablets, select yeast extract, tryptone, agar, Tween 80, and l-histidine dihydrochloride were purchased from Sigma-Aldrich; acetic acid, sodium chloride p.a., and n-hexane 99% p.a. from Penta Chemicals; sodium tripolyphosphate (TPP) from Acros Organics; ethanol 99.8% for UV/VIS spectroscopy, and glycerol anhydrous g. r. from Lach-Ner; curcumin 99.9% from Chromadex; Brain–Heart-Infusion broth (BHI, Hirn-Herz-Glucose-Bouillon für die Mikrobiologie) from Carl Roth; sunflower lecithin from Fichema s.r.o. Materials for finger replica preparation—FIMO^®^ soft oven-bake modelling clay and liquid silicon paste with a hardener were purchased from Staedtler.

Antimicrobial foam soap was purchased from Tork. Chlorhexidine digluconate (CHG) 25% w/w aqueous solution was provided by Zentiva, k.s. Two commercially available sanitisers used as a positive control for bacterial testing were purchased in a local store, i.e., ethanol-based gel (72% w/w) from Sanytol and 2% CHG alcoholic solution (IPA 70% v/v) from B. Braun Medical AG.

### Antiseptic particles preparation

Chitosan particles containing chlorhexidine digluconate (CHG) as an antiseptic were prepared by spray drying. The feed solutions for spray drying were prepared as 0.5% w/w chitosan solution with added CHG equal to 0, 1, 5, or 10% of the relative mass fraction defined as the weight of CHG per dry weight of chitosan. First, a defined amount of chitosan powder was added into demineralised water under constant stirring (1400 rpm, 40 °C) to form an aqueous suspension. Then, acetic acid was added dropwise to give a 1% v/v solution. The suspension was stirred for 30 min at 40 °C for complete chitosan dissolution. After cooling to room temperature, CHG was added, and the solution was thoroughly homogenised.

Next, a different amount of ionic cross-linker TPP was used to alter spray-dried chitosan carriers' solubility/swelling properties. The relative mass fraction of TPP with respect to chitosan was chosen as 0, 4, 8, and 16%. TPP dissolved in 10 mL of 1% v/v acetic acid was added dropwise by a syringe into the vortex of stirred chitosan solution to prevent excessive gelation and formation of agglomerates, which may cause clogging of the atomising nozzle during spray drying.

Using an ultrasonic nozzle (cat. no. 110 599 80), the homogenised solutions were spray dried by BÜCHI Mini Spray Dryer B-290 in open mode configuration, i.e., drying air is not recycled. The nozzle voltage was set to 5.7 V, the feeding rate to 5 mL/min, the drying air temperature at the inlet was maintained at 120 °C, and the aspirator rate was kept constant at 31.5 m^3^/h (90% of the maximum flow rate). Particles collected via high-performance cyclone were kept for a week under reduced pressure in a desiccator to remove any remaining traces of unbound moisture and acetic acid. The final powder product was stored in sealed glass containers at room temperature, protected from direct sunlight. Blank chitosan particles (used as a negative control for antibacterial assays) were prepared following the same procedure, except no CHG was added during the preparation.

For testing of particle adhesion, CHG was replaced with curcumin dye, which emits fluorescence upon excitation by visible light. These particles were prepared following the same protocol, with the curcumin content of 1% w/w in the final dry product. The relative mass fraction of TPP was 4% w/w_chitosan_. This amount of TPP cross-linker was used in all fingerprint assays.

### Particle characterisation

#### Yield and encapsulation efficiency

The powder yield was calculated as the percentual mass fraction of collected particles divided by the theoretical yield based on feed composition and the overall atomised amount of feed solution. Particles were weighed after one week of storage under reduced pressure to ensure no unbound water or acetic acid was present in the particles.

Encapsulation efficiency was evaluated spectrophotometrically utilising SPECORD 205 UV/VIS spectrophotometer. The powder sample (20 mg) was sonicated for 10 min in 200 µL of ethanol for UV/VIS spectroscopy, transferred in 5 mL of deionised water, centrifuged, and the absorbance of the supernatant was measured at 260 nm. The measurement was performed simultaneously for chlorhexidine-loaded and blank particles of the same carrier composition. Therefore, the absorbance of the blank particles could be subtracted as background from CHG particles to eliminate any possible contribution of dissolved chitosan and TPP. The final concentration of CHG was calculated from the calibration curve. The encapsulation efficiency and drug loading of CHG are presented as mean ± SD, n = 3 in all cases.

### Particle size distribution and morphology

Particle size distribution (volume-based) was determined with Horiba Partica LA-950 S2. Particles were dispersed in 99.8% ethanol for UV/VIS spectroscopy and sonicated for 10 min before measurement to break down any temporary aggregates. Mean size and span defined as (D_90_ − D_10_)/D_50_) were calculated to describe the PSD of prepared samples.

Particle surface morphology and shape were examined with a scanning electron microscope (SEM) Jeol JCM-5700 using an accelerating voltage of 5 kV. Before the measurement, samples were attached to aluminium stubs with a conductive carbon double-sided adhesive tape and sputter coated with a gold layer with the Emitech K550X sputter-coater at a current of 25 mA for 3 min.

### Chlorhexidine release

Time-dependent release of chlorhexidine from spray-dried particles into a phosphate-buffered saline (PBS) was measured with UV/VIS spectrometer SPECORD 205. First, a particle sample of 10 mg was placed into an envelope folded from filter paper. Then, the envelope was attached to a holder created from a syringe plunger and placed into a quartz cuvette with a magnetic stirring bar, as shown previously^21^. In such a configuration, the particle sample was physically separated from the surroundings, ensuring that no particles would interfere with the device's lightpath and that the mass transfer of solutes to and from the sample should not be affected by the presence of a highly permeable paper wall. Finally, 2.5 mL of PBS was added, the cuvette was sealed, and the absorbance at 260 nm (A_260_) was immediately measured (time 0 min). The cuvette was placed on a magnetic stirrer, and A_260_ was measured in 1.5-min intervals until 15 min, 5-min intervals until 2.5 h, and finally after 20, 22, and 24 h from the beginning of the measurement. The concentration of CHG in the solution and the absolute amount of released CHG were calculated from measured absorbance values. Based on the calculated encapsulation efficiency values for every sample, the relative amount of released CHG in time was determined. The measurement was conducted three times for every sample.

### Finger-replica preparation and testing

A wearable human skin replica suitable for particle deposition and adhesion studies was proposed and tested. The preparation was divided into two steps. First, a negative imprint was obtained by pressing an index finger into a preheated and kneaded block of FIMO (commercial polymeric material based on polyvinyl chloride). The imprint was thermally hardened in an oven at 110 °C for 30 min. The quality of the final imprint was inspected under SEM.

**Figure 1:**
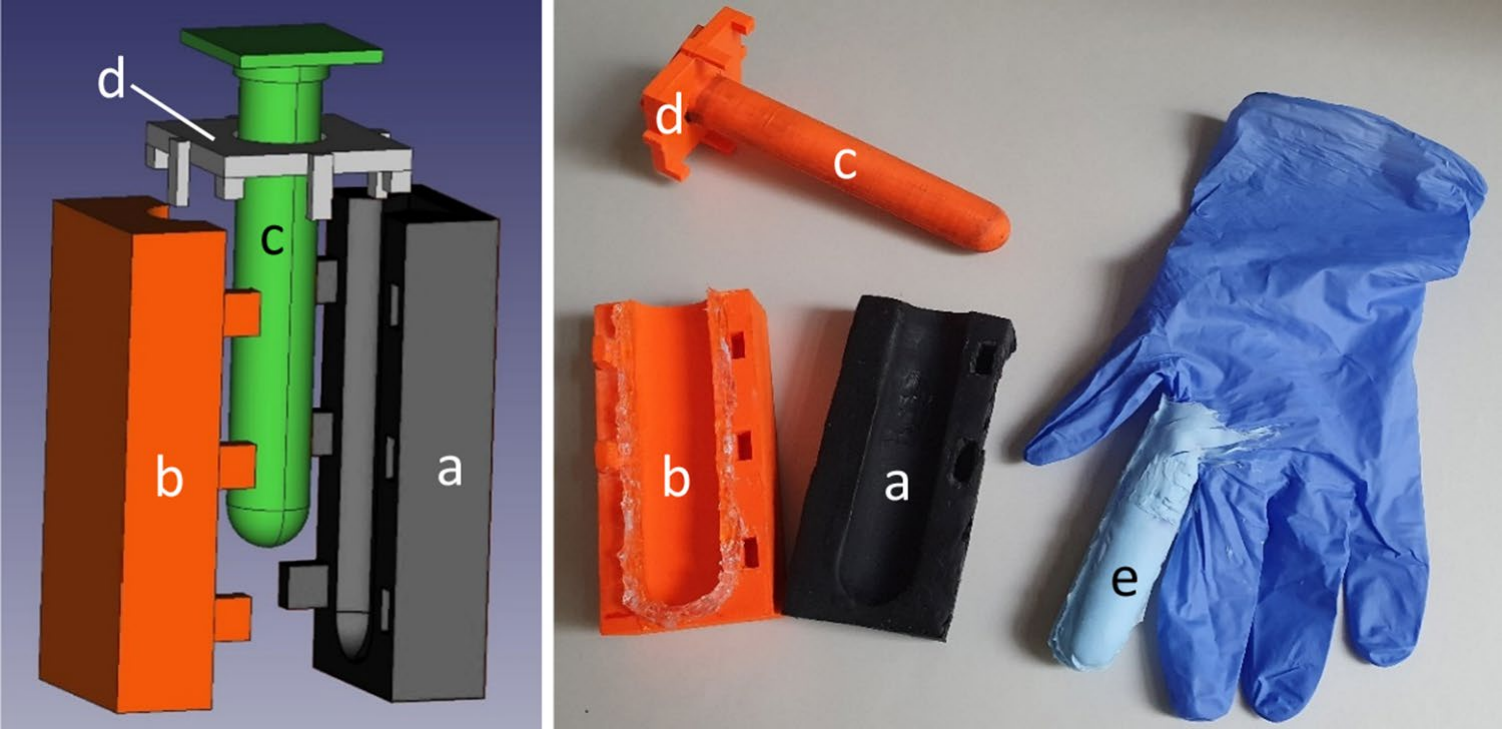
Finger replica—3D model of form for preparation (left), and an actual replica (right); (**a**) negative replica of papillary ridges, (**b**) the second part of the outer form, (**c**) insert for a glove, (**d**) connecting piece, (**e**) positive replica of papillary ridges (light blue) attached to nitrile glove.

In the second step, the skin pattern was transferred onto a nitrile glove using a 3D printed mold designed in AutoCAD (Fig. 1). It consists of four parts: the negative replica with an imprint (a), the second outer part of the form determining the shape of the prepared artificial finger (b), the insert part allowing for insertion of the index finger of nitrile glove (c), and a connecting piece fixing the insert in the correct position (d).

In the first step, parts (a) and (b) were press-fitted together, and the inner pocket space was filled with silicon (purchased from Manumi) mixed with a hardener. Part (c) was put in a finger of a nitrile glove and inserted in the (a, b) form using part (d) as a guide. The size of the nitrile glove was chosen to fit the original model. The whole assembly was secured with C-clamps and left to harden for two days. After curing, it was disassembled, and the positive replica (e) was carefully peeled off. The form (a) with the negative replica showed no visible signs of degradation or wear, allowing repeated use.

The acquired finger replica was first observed with a scanning electron microscope Jeol JCM-5700 to double-check the quality of the pattern. Approximately 1 cm^2^ of the replica was attached to an aluminium stub with carbon double-sided adhesive tape. Further sample preparation and setting conditions were the same as for the particle samples. The width and height of the imprinted papillary lines and ridges were estimated via the Keyence VR-5000 profilometer and compared with values found in the literature. The finger replica was cut in half before measurement, so the part with the imprinted papillary lines could be flattened.

### Deposition and abrasion studies of the particles

#### SEM—qualitative study

Particle spatial deposition on the fingerprint replica immediately after the application was examined by SEM. Particles were applied uniformly over the finger replica using a cotton swab, followed by rubbing over another replica, mimicking the intended real-life application. For the analysis, a 1 cm^2^ piece of fingertip area was cut off, attached to the aluminium stub, and gold sputter coated.

For the abrasion study, finger replicas (attached to nitrile gloves) with applied particles were used by six volunteers for 1-h carrying out common activities such as interaction with miscellaneous objects, e.g., doorknob, keyboard, mouse, or pen. The only temporary prohibited activity was hand cleaning. After the elapsed time, a sample of the glove from the fingertip area was cut off and examined. SEM images were manually coloured in Adobe Photoshop for better visibility.

### UV/VIS spectroscopy—quantitative study

As the SEM only provides information on the spatial distribution of particles, it cannot access information about the total amount of deposited particles in multiple layers. Thus, a method for a quantitative evaluation of the adhesion and abrasion on the volunteers' hands was developed using particles with 1% w/w curcumin. Chlorhexidine particles were not suitable since substances on our hands, e.g., sebum, absorb light of similar wavelengths as studied chlorhexidine and strongly interfere with the spectroscopic measurement.

A square of bare skin (1 cm^2^) was outlined on a finger using adhesive tape, so the dimensions of the covered area were well-defined. Curcumin-loaded particles were applied to this area, and fingers were rubbed gently against each other for consistent application. Afterwards, the adhesive tape was peeled off, and adhered particles were transferred into absolute ethanol. The emission was measured at 550 nm (when excited with 434 nm light) using TECAN Infinite 200 PRO microplate reader. Finally, the amount of particles adhered to 1 cm^2^ of skin was calculated, assuming a uniform distribution of curcumin in spray-dried particles. The same procedure was followed with the silicon finger replicas.

For the abrasion study, particles were applied following the same protocol. However, after the application, adhesive tape stayed attached, and volunteers participating in the test conducted everyday activities. After a given time (30 min/1 h/2 h), adhesive tape was removed, and the remaining particles were transferred to ethanol, and the amount of particles was calculated using the emission measurement described before. The test was performed in the same way with silicon finger replicas. The abrasion test was carried out six times with different volunteers and three times with finger replicas. For each volunteer, the amount of adhered particles at 0 min was determined before the abrasion testing. This approach was needed since the adhered amount varied substantially among volunteers, and even the same subject showed various amounts of adhered particles on different days when air temperature/humidity and skin condition changed.

Since the activities performed during the abrasion study were diverse, another set of experiments was conducted to describe particle transfer to the surface under well-defined conditions. The index finger (real or replica) with a defined amount of applied particles was pressed on a glass coverslip, allowing for straightforward visualisation. This procedure was repeated 12 times consecutively on a new clean surface under similar conditions, i.e., finger pressure, contact duration, and direction of approach. The particle coverage area was evaluated from binarised microscopy images by Fiji software.

### Bacterial tests

#### Disc/well diffusion tests

Disc and well diffusion tests were conducted on two different bacterial species, gram-negative *Escherichia coli,* and gram-positive *Staphylococcus epidermidis*, to compare the bacteriostatic effect of prepared particles and commercial sanitisers. Luria–Bertani (LB) agar plates for *E. coli* cultivation were prepared as follows: 5 g tryptone, 5 g sodium chloride, 2.5 g yeast extract, and 7.5 g agar were mixed in 0.5 L of demineralised water and autoclaved at 121 °C for 20 min. After cooling down to 60 °C, 18 mL of LB was poured into 90 mm diameter Petri dish. Petri dishes were stored upside down in a refrigerator to prevent contamination and excessive water condensation. For *S. epidermidis*, brain–heart-infusion (BHI) agar plates were prepared from 18.5 g of BHI broth and 7.5 g of agar in 0.5 L of demineralised water. The rest of the procedure was performed in the same way.

Agar plates inoculated with bacterial suspension OD_600_ = 0.4 were left to dry for 10 min. Next, either paper discs (d = 6 mm) were gently pressed on the agar, or holes serving as wells (d = 6 mm) were punched. For particle testing, 10 mg of particles was placed into each hole topped with 120 μL of demineralised water. When commercial sanitisers (ethanol-based gel and CHG solution) were tested, 40 μL of each was pipetted on a paper disc. As a positive control, 40 μL of kanamycin solution (50 mg/mL) was used. Plates were incubated at 37 °C for 20 h, and the diameter of inhibition zones was measured using Fiji software^25^. Tests were conducted in triplicates, and data were analysed by one-way ANOVA followed by Tukey post hoc test.

### Finger imprint assay

Testing of the prolonged effect of antiseptic particles was inspired by Balkrishna et al.^26^. The volunteer’s hands were thoroughly washed with an antimicrobial foam soap (Tork), rinsed with tap water, and dried with lint-free paper towels. On the dominant hand of each volunteer, CHG particles were applied as a uniform layer with a brush. To increase testing consistency, the same person performed this part of the procedure during all tests. The non-dominant washed hand served as control or was treated with a commercial sanitiser (72% alcoholic gel, 2% CHG solution) or blank chitosan particles. Subjects participating in testing were allowed to conduct any activities except for hand rubbing, washing, or the additional application of any sanitiser. After the specified time (30 min/1 h/2 h), fingers were gently pressed on a BHI agar plate (a general-purpose nutrient medium) with the chlorhexidine inactivators. For purposes of the finger imprint assay, it was essential to inactivate any chlorhexidine (free or encapsulated in particles) transferred along with pathogens from the finger to the agar plate. Chlorhexidine inactivation was achieved by adding 0.526 g l-histidine dihydrochloride, 1.578 g sunflower lecithin, and 15.78 g Tween 80 to BHI stock solutions following previously reported protocol^27^. Plates were incubated at 37 °C for 20 h, and bacterial/fungal growth was visually evaluated. For each test duration, 1, 5, and 10% CHG particles (4% TPP) were tested against non-disinfected hands four times. Blank particles and commercial sanitisers were tested four times for 30 min and 1 h. After the incubation period, the growth of colonies on the plates was compared.

All participants involved in the testing process were given full disclosure about the nature of the substances used. Prior to the testing, each participant signed an informed consent document. The proposed procedure and processing of solution results are in accordance with the ethical principles of UCT Prague (Approval Number EK3/24), established by the Code of Ethics of UCT Prague dated 27/09/2018 and with the principles of the Declaration of Helsinki (Ethical Principles for Medical Research Involving Human Subjects).

## Results and discussion

### Particle characterisation

#### Powder yield, encapsulation efficiency and loading degree

The powder yield, encapsulation efficiency (EE) and loading degree (wt% of CHG in the powder) for each particle type are summarised in the following chapter.

 The powder yield was within the range of 42–56% for all formulations (Table 1). With increasing CHG and fixed TPP content, the yield decreased. On the contrary, the yield was improved with higher TPP and fixed CHG content. Particle losses were caused by the deposition of atomised solution on the walls of the drying chamber and imperfect separation in a cyclone, leading to the loss of the sub-micron-sized particles in exhaust air.

**Table 1:** Powder yield, encapsulation efficiency and loading degree for all particle types.

Particle type	Yield (%)	Encapsulation efficiency (%)	Loading degree (%)
1% CHG 0% TPP	50.38	77.5 ± 2.5	0.77 ± 0.02
1% CHG 4% TPP	52.11	82.9 ± 1.7	0.79 ± 0.02
1% CHG 8% TPP	53.51	94.5 ± 2.3	0.87 ± 0.02
1% CHG 16% TPP	56.03	63.1 ± 5.5	0.54 ± 0.05
5% CHG 4% TPP	45.12	72.7 ± 2.5	3.38 ± 0.12
10% CHG 4% TPP	42.96	74.4 ± 1.8	6.68 ± 0.17

The encapsulation efficiency of CHG varied significantly from 63 to 95%. The lowest EE was found for particles with the highest TPP amount (16%). This phenomenon is probably caused by the interaction of unbound TPP (negatively charged phosphate groups) with positively charged CHG, leading to precipitation. Moreover, the interaction of CHG with negatively charged phosphate groups in buffers (PBS) significantly impacts the CHG release kinetics, which is discussed in the following chapter.

### Size distribution

The volume-based size distribution of all samples determined by Horiba Partica LA-950 S2 is summarised in Table 2. The PSDs of spray-dried particles were unimodal and narrow, with a span index of 0.9 for all samples. The similarity of PSD of all samples is given by the same spray drying conditions, used equipment, and properties of an atomised solution. With a higher TPP amount, the mean particle diameter increased for fixed CHG content. No particular trend was observed for increasing CHG content in the final formulation.

**Table 2:** Particle size characteristic of all samples.

Particle type	Mean size (μm)	Span index
1% CHG 0% TPP	6.3 ± 2.1	0.9
1% CHG 4% TPP	6.3 ± 2.2	0.9
1% CHG 8% TPP	6.6 ± 2.3	0.9
1% CHG 16% TPP	7.7 ± 2.8	0.9
5% CHG 4% TPP	5.6 ± 2.0	0.9
10% CHG 4% TPP	6.2 ± 2.2	0.9

### Particle morphology

**Figure 2:**
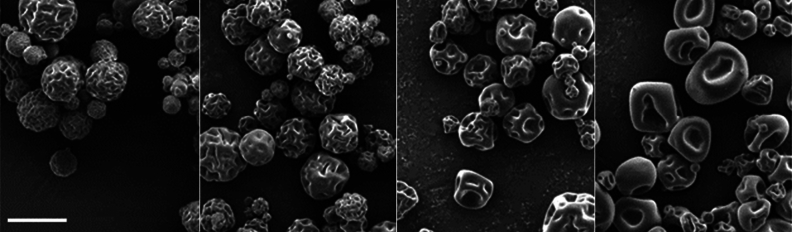
Spray-dried particles with 1% CHG and different amounts of crosslinker—from left to right: 0, 4, 8, and 16% TPP; the scale bar represents 10 μm for all images.

**Figure 3:**
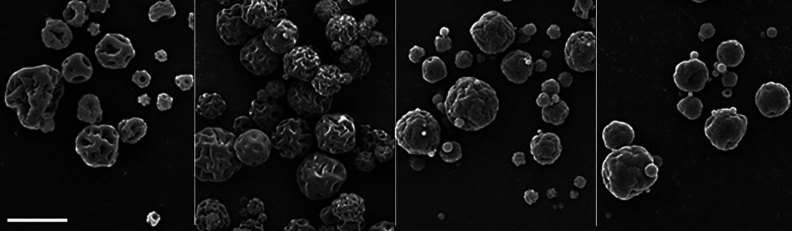
Spray-dried particles with 4% TPP and different amounts of CHG—from left to right: 0, 1, 5 and 10% CHG; the scale bar represents 10 μm for all images.

Spray-dried chitosan particles are hollow spheres with characteristic brain-like surface topology (see Figs. 2 and 3). The hollow structure is typical for skin-forming carrier materials such as chitosan. As the hot gases inside of the chitosan shell cool down, the internal pressure decreases, causing skin shrinkage followed by the formation of grooves and folds. Higher surface roughness and irregular particle shape can benefit topical application, increasing the particle surface area and positively promoting particle–surface and interparticle interaction and particle retention.

The rigidity of the chitosan shell and the dimensions of the grooves strongly depend on the extent of cross-linking of chitosan chains by TPP. With the higher TPP content, the shell’s rigidity increases, reducing the number of grooves but increasing their respective size and shape irregularity (Fig. 2). On the other hand, a higher CHG amount resulted in smoother surface topology and higher sphericity of collected particles (Fig. 3).

### Chlorhexidine release

The release of CHG from spray-dried particles (10 mg) was studied using PBS buffer, reflecting that human sweat contains electrolytes that strongly affect CHG solubility^28^. The usual range of electrolyte values in sweat in adults is up to 70 mM/L of Na^+^ and 55 mM/L of Cl^−^^29^.

**Figure 4:**
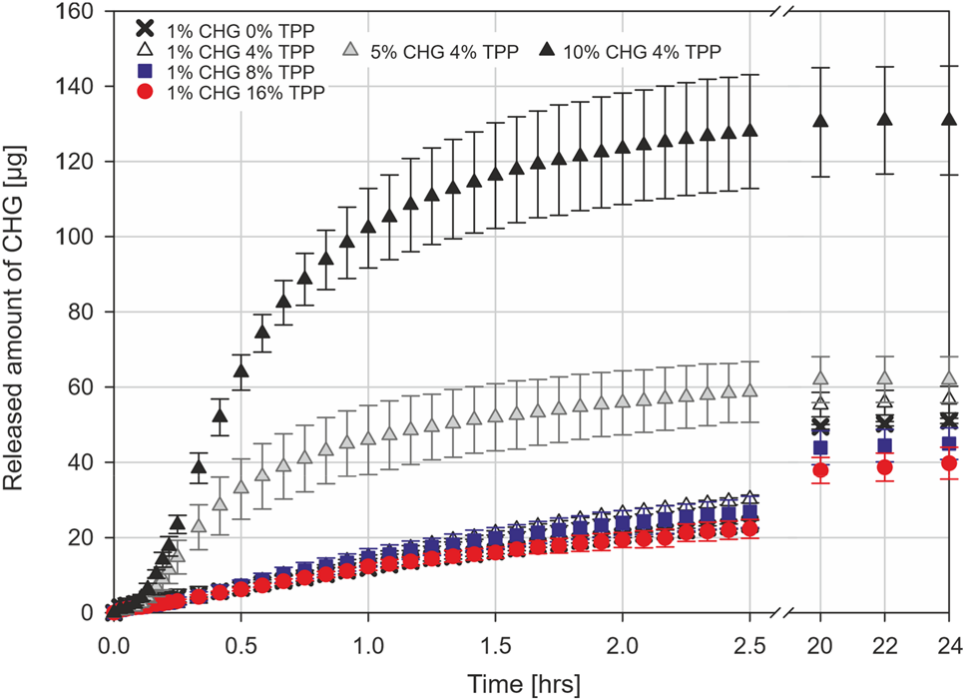
Release of CHG from spray-dried chitosan particles (all tested combinations of CHG and TPP as described in Table 1) in PBS buffer.

**Figure 5:**
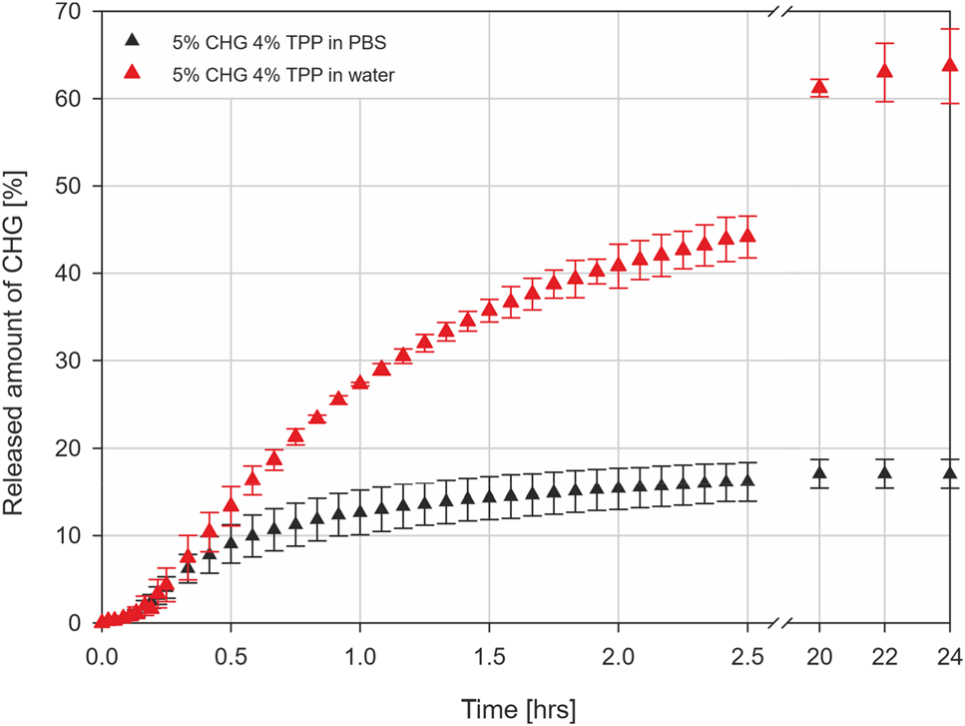
Release of CHG from spray-dried chitosan particles (5% CHG and 4% TPP) in PBS and demineralised water.

The absolute amounts (in µg) of released CHG from all types of particles are shown in Fig. 4, and the calculated relative amount (in %) based on encapsulation efficiency is shown in Fig. SI 1. The initial lag phase is caused by the wetting of dry particles in the envelope. 1% CHG particles with various TPP content showed similar dissolution profiles during the first 2 h when 19 µg (16% TPP) to 26 µg (4% TPP) of CHG was released. Based on previously determined EE, the relative amount of dissolved CHG after 2 and 24 h ranged from 26 to 35% and 55 to 73%, respectively. Increasing the concentration of CHG from 1 to 5% and 10% resulted in approx. 3-times and 6-times higher amounts of released CHG after 2 h, which is the longest considered duration for skin application. The released amounts for 5% and 10% CHG particles remained unchanged for the next 24 h even though the solubility limit of CHG in PBS was not yet reached. This effect can be explained by the non-trivial interaction of CHG with negatively charged TPP, phosphates, and NaCl in PBS buffer. To demonstrate the influence of buffer choice, release kinetics for 5% CHG particles in water and PBS were compared (Fig. 5). The initial lag phase of the same duration is present for both water and PBS. However, the release amount of CHG in water after two and 24 h is 2.75 and 3.82 times higher than for PBS, and the plateau of the dissolution profile has not been reached.

### Fingerprint replica

The fingerprint replicas with imprinted papillary lines served as an adequate alternative to human skin, which can be worn and analysed afterwards. Silicon was selected as the most suitable material for faithful replication of finger topology because of its flexibility, durability, lack of antibacterial properties, and simplicity of sterilisation. Other materials, i.e., NOA81, PDMS or polyvinyl acetate, were considered, tested and eventually rejected when one or more desired specifications of the final replica were not met.

**Figure 6:**
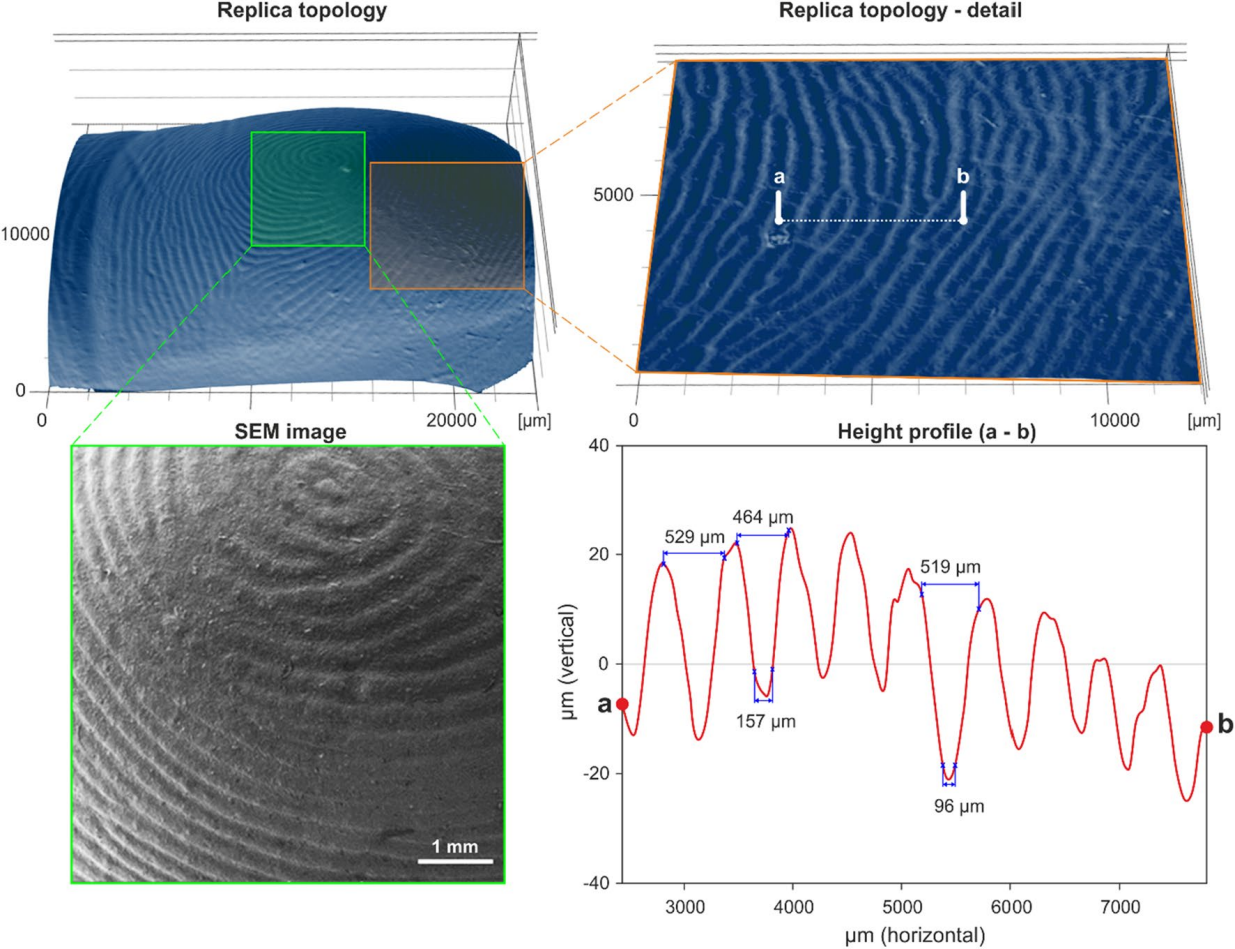
Visualisation and analysis of replica surface by SEM (grayscale image in the bottom left corner) and optical profilometry (images on the top row and analysed height profile in the bottom right corner).

SEM and optical profilometry confirmed the successful replication of papillary lines and ridges of the donor (Fig. 6). Optical profilometry confirmed that both height (20 to 40 µm) and pitch of papillary ridges (~ 500 µm) were in agreement with previously reported data^30,31^. Most importantly, the particle adhesion to replica and human skin was comparable, as described in the following section.

### Particle adhesion and abrasion

#### Qualitative study—SEM

**Figure 7:**
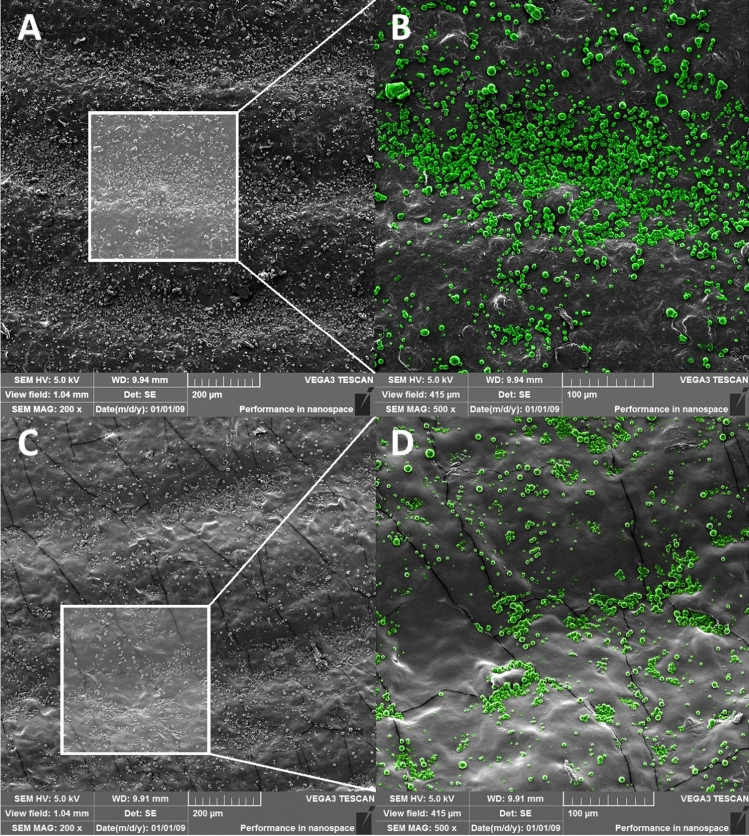
Particle adhesion on a finger replica (4% TPP) immediately after application (**A**,**B**) and after 1 h of ordinary activities (**C**,**D**). Particles in zoomed insets were artificially coloured in Adobe Photoshop for better visibility.

For the study of particle adhesion, a small amount of 4% TPP particles was uniformly spread across the finger replica, and the excess was removed by gentle hand rubbing. After the first interaction with the foreign surface, a significant amount of particles (previously located on top of the ridges) was detached and transferred to the surface. The remaining particles were preferentially located in less exposed valleys partially protected against mechanical abrasion. The particle coverage of the finger replica immediately after application and 1 h of wear is shown in Fig. 7. Initial particle distribution confirms that particles are located mainly in valleys serving as particle deposits. After 1 h of replica wearing and interacting with various objects, a high amount of particles remained still attached to the replica surface, encouraging the use of particles for a prolonged time.

### Quantitative study—UV/VIS spectroscopy

**Figure 8:**
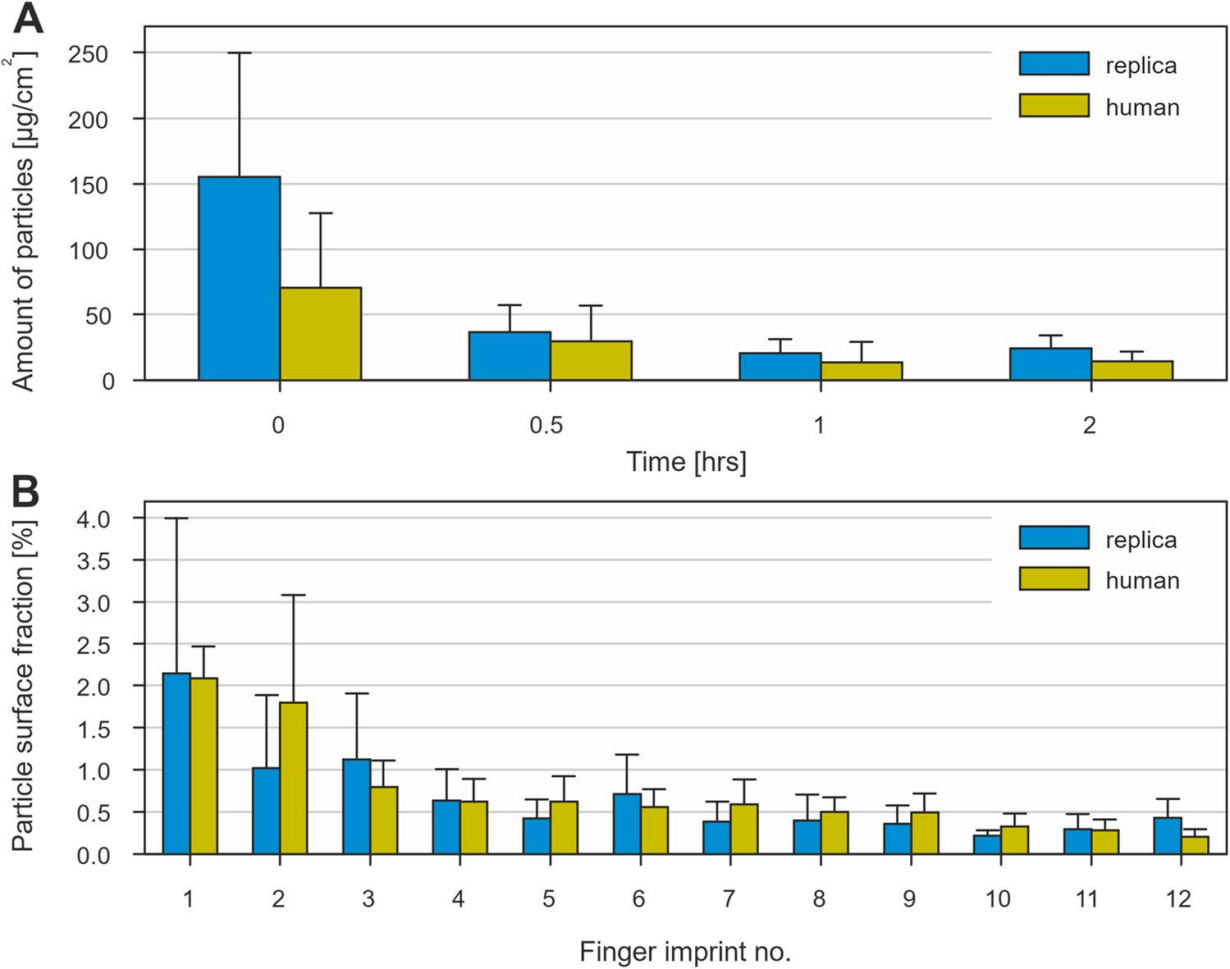
Quantitative comparison of finger replica with human finger: (**A**) time dependence of the amount of adhered particles; (**B**) particle deposition on glass substrate upon repeated imprints.

The comparison of the adhered amount of particles (µg/cm^2^) in the fingertip area of the replica and volunteers is shown in Fig. 8A. The experiment duration was set to 0, 0.5, 1, and 2 h, based on the intended duration of antibacterial testing. The initial amount of adhered particles varied significantly since multiple factors such as room temperature, air humidity, skin condition, or the rubbing force during application all affect particle adhesion. On the defined 1 cm^2^ area of the volunteer’s fingertip, 70 ± 57 μg of particles was attached (t = 0 h). When the same protocol was conducted with the silicone finger replica, 156 ± 94 μg of particles was applied. More than twice as high amount of adhered particles was most probably caused by human factor, since volunteers had a tendency to rub their hands more gently with silicon fingers attached to gloves on their own hands.

After 0.5 h of common everyday activity, more exposed and loosely adhered particles detached, and only 30 ± 27 μg/cm^2^ and 37 ± 21 μg/cm^2^ particles remained on the human skin and the silicone replica, respectively. These values are comparable (in contrast to initial adhered amounts), which can be interpreted by the fact that most exposed particles were detached, resulting in a similar particle distribution and surface coverage. The most important finding is that a significant amount of adhered particles was detected even after 1 and 2 h. This leads us to conclude that the particle fraction remaining attached after the first hour can provide prolonged protection until the actives are depleted or mechanically removed, e.g., by hand washing or sweating.

Figure 8B shows particle coverage of the glass substrate upon repeated interaction with the replica and real index finger with applied particles. As expected, the highest amount of particles was transferred to the glass surface with the first imprint. With every following interaction, the transferred amount was slightly reduced up to the fourth imprint when the transferred amount stayed for both replica and silicon skin comparable. Finger imprints on the glass shared the same patterns given by the finger topology, suggesting that most of the transferred particles were initially located on top of friction ridges, which agrees with SEM images in Fig. 7.

According to an adhesion study, the amount of particles applied initially varied considerably due to various factors mentioned earlier. Nevertheless, after 30 min, similar average amounts of particles per area were observed on both human skin and silicon replica. This was because loosely attached particles were either detached or redistributed after multiple contact with foreign objects. These findings indicate that silicon artificial skin can be, with caution, used as a topologically true substitute for human skin in adhesion and attrition studies where direct analysis is difficult or impractical, but the issue of higher reproducibility of powder application needs to be addressed first.

### Antibacterial effect

#### Disc/well diffusion tests

**Figure 9:**
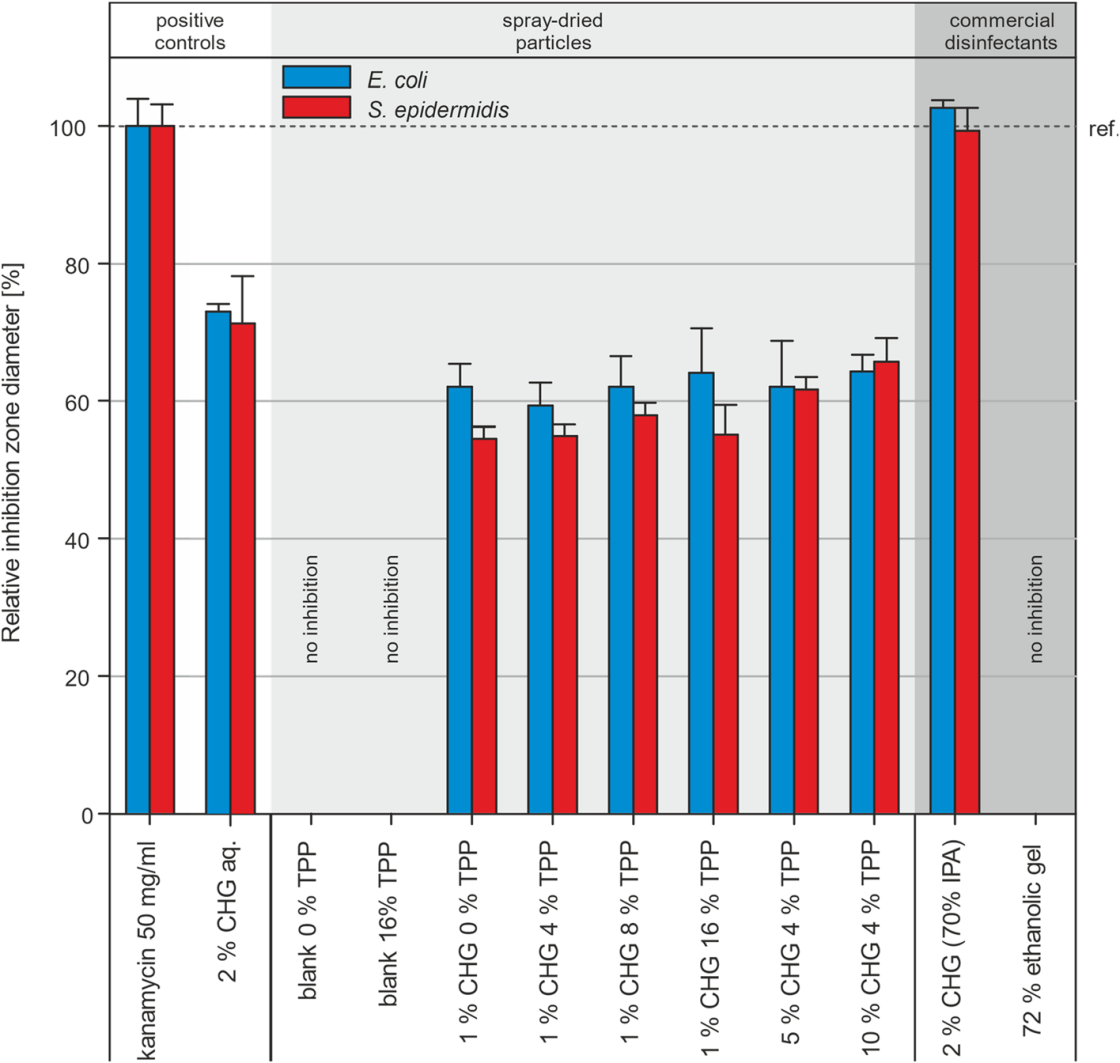
Antibacterial efficacy (based on inhibition zone from agar plates) of all particle types (10 mg) and commercial sanitisers (40 μL). Kanamycin (positive control) is taken as a reference (100%).

Disc tests (for liquids) and well diffusion tests (for particles) were conducted to confirm the bacteriostatic effect of prepared powders and commercial products. All results are summarised in Fig. 9. 1% CHG spray-dried particles with various TPP contents showed similar effects for both bacterial strains. Increasing the CHG loading by five times did not lead to any improvement in the results. However, particles with the highest CHG content, i.e., 10% w/w_chitosan_, showed a modest but statistically significant increase in observed antibacterial effect against *S. epidermidis* compared to 1% CHG particles. No inhibition zones were observed for blank chitosan particles with or without TPP for both bacterial strains. The similar effect observed for all types of particles with varying CHG content can be attributed to the fact that the reported MIC values for *E. coli* and *S. epidermidis* are less than 2 µg/mL^32,33^. Therefore, wetting 10 mg of the sample with 120 μL of water would result in CHG concentration two orders of magnitude higher, even for the sample with the lowest CHG content. As a result, the size of inhibition zones is determined by the amount of added solvent rather than the concentration of CHG.

Next, two commercial sanitisers, an ethanol-based gel and a 2% CHG alcohol solution, were compared for their antibacterial activity. Ethanol-based gel could not soak into a paper disc before ethanol evaporated, resulting in the absence of inhibition zones. When the CHG alcohol solution was used, the inhibition zones were comparable to the kanamycin reference, which is given by the simultaneous presence of two actives (IPA and CHG) with different mechanisms of action. Aqueous 2% CHG solution (not a commercial product) used as the second control inhibited the growth of both bacterial strains significantly less than 2% CHG alcohol solution. It is important to note that disc diffusion tests, which use solutions, and well tests, which use particles, cannot be directly compared. Moreover, CHG must be released from particles and consequently transported to an agar surface, which is not required for CHG solutions.

Although these tests help to compare the antibacterial performance among particles and solutions in a controlled environment, they do not provide relevant information about the extent and duration of hand protection in real-life scenarios, which is critical for meaningful comparison with products available on the market. For this reason, a series of tests with twelve volunteers was conducted, which will be described in the following section.

### Finger imprint assay

Twelve volunteers affiliated with UCT Prague kindly agreed to participate in antibacterial activity testing. The final results are summarised in image arrays, where photographs of agar plates with imprinted fingers of all volunteers are arranged. Typically, four to five fingers were imprinted depending on hand size. For better visibility, all images were converted to inverted 8-bit grayscale, and brightness and contrast were adjusted. This form of visualisation was selected to quickly compare the amount and type of colonies that grew after incubation. Smaller round-shaped colonies with a clear border belong to bacteria or yeasts, which can overlap if their number is too high, preventing their counting. The more colonies are present, the smaller colonies in terms of size are formed as growth nutrients are consumed. One grown colony represents one bacterial or yeast cell transferred to the agar. The larger, irregular areas are typically covered with moulds.

**Figure 10:**
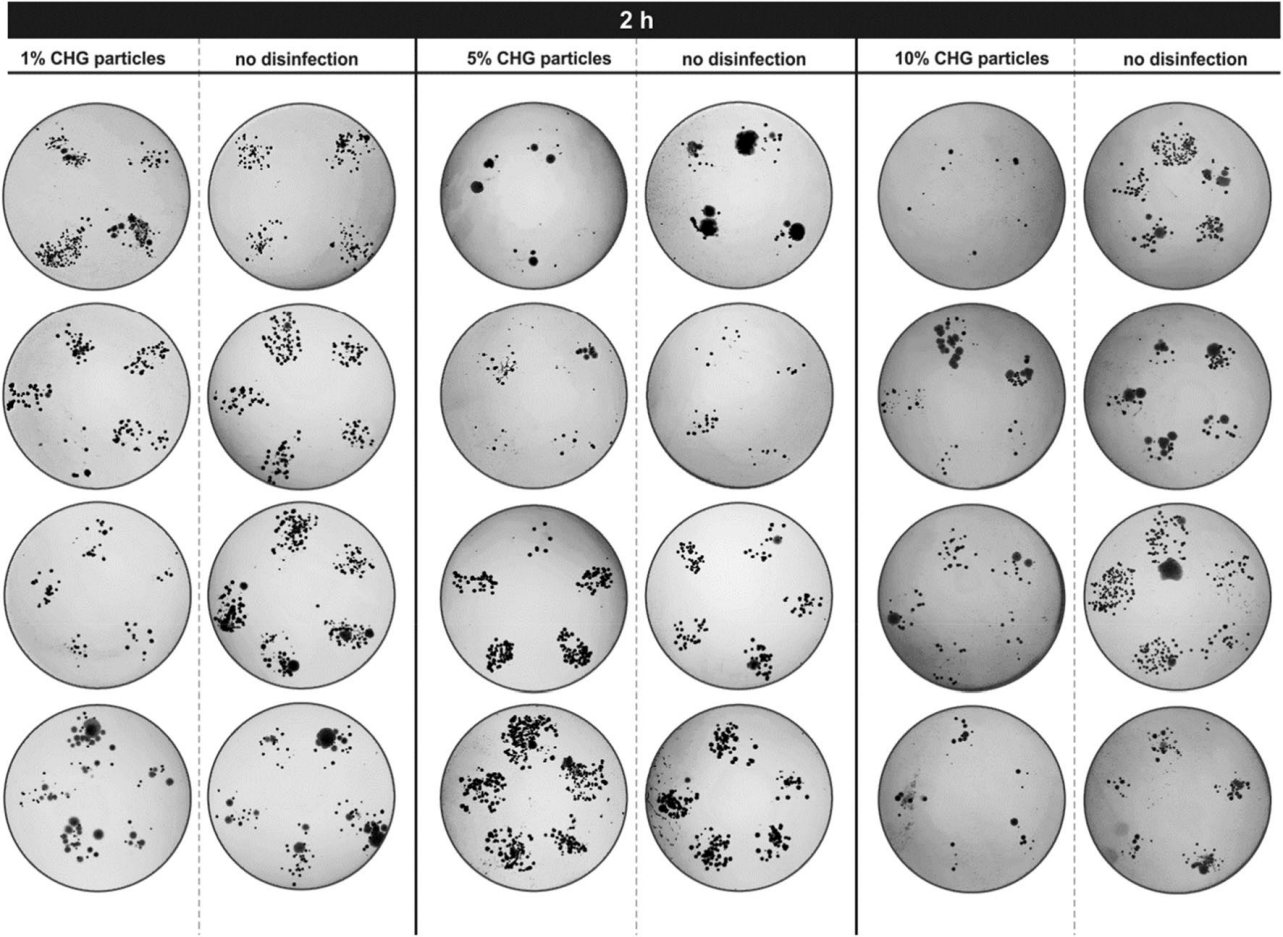
Finger imprint assays—the efficacy of spray-dried particles (1, 5, and 10% CHG) 2 h after application.

The antimicrobial effect of 1, 5, and 10% CHG particles was analysed after 30 min, 1 h and 2 h after application. The results of finger imprints after the longest time period are shown in Fig. 10. After 2 h, particles with 1% and 5% CHG showed little antimicrobial effect compared to hands without any treatment. Particles with the highest CHG concentration (10%) were able to reduce the number of emerging colonies, which can be observed in the last column of Fig. 10. Even though the lowest concentration was not fully effective after 2 h, it showed significant antimicrobial activity after 1 h (data for 1% CHG shown in Supplementary information, Fig. SI 2). The additional experimental results with 1% and 10% CHG particles after 0.5 and 1 h are shown in Figs. SI 2 and SI 3. It should be noted that CHG particles were consistently applied to the participant's dominant hand, where the risk of contamination is higher, and the possible effect of transferred CHG to BHI agar plates was neutralised by the presence of CHG inhibitors in the growth medium.

**Figure 11:**
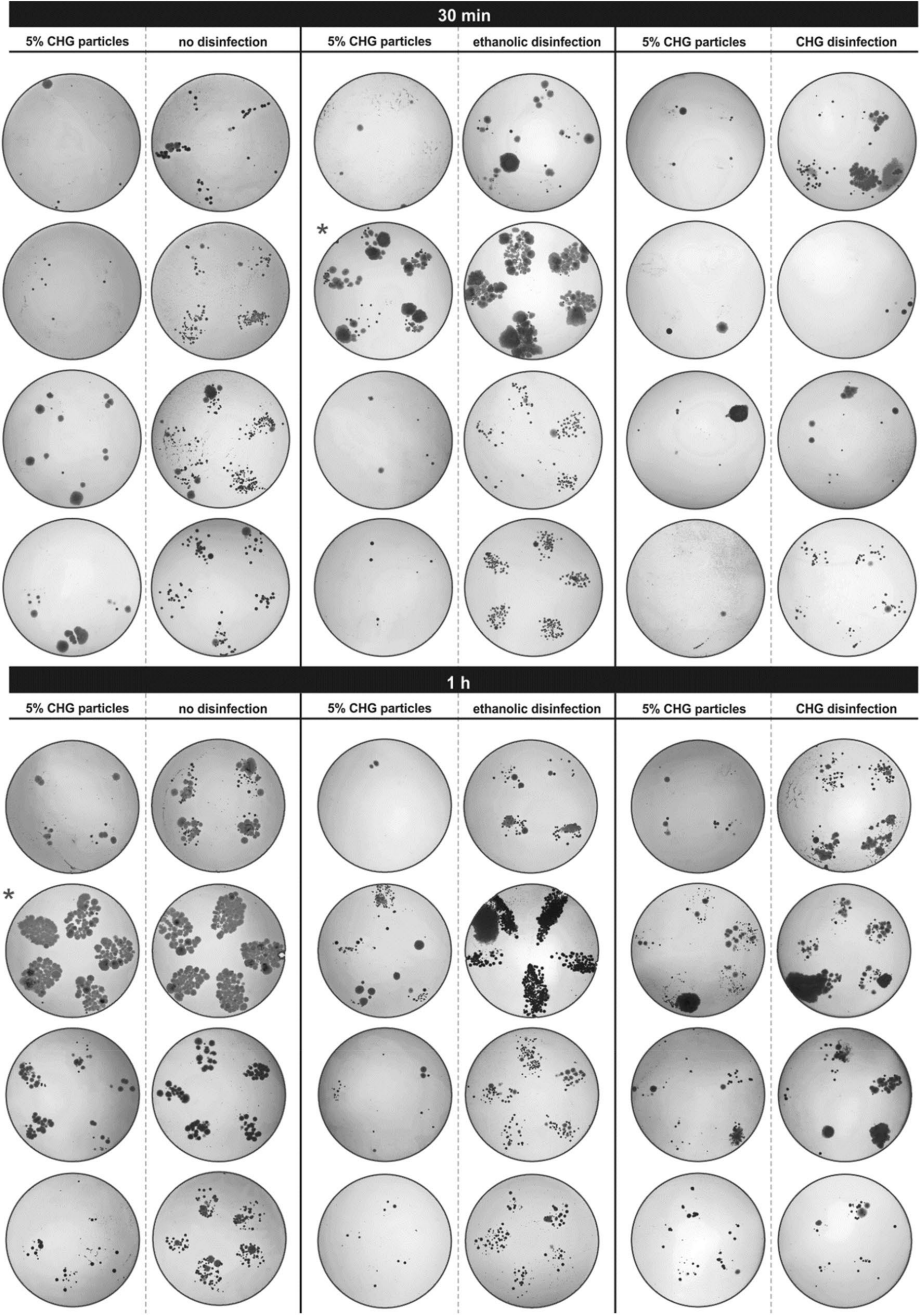
Finger imprint assays—the efficacy of 5% CHG particles and commercial sanitisers after 30 min (upper array) and 1 h (bottom array) after application.

In contrast to commercial sanitisers, CHG particles applied on soap-washed dry hands must be partially hydrated, e.g. by sweat, to release encapsulated content. Thus, the question remains whether such particles can release a sufficient amount of CHG in a shorter time (30 min) to provide detectable skin protection. Preliminary tests confirmed that after 30 min 1% CHG particles were effective only for 50% of volunteers (Fig. SI 2). On the other hand, 1 h after the application, only low bacterial counts were observed in all cases, which provides an effective time frame for 1% CHG particles. Referring to the release kinetics in Fig. 4, the expected amount of released CHG in 30 min was, for fully hydrated 5% CHG particles, approx. four times higher than for all variants of 1% CHG particles. For this reason, the higher concentration of CHG (5%) was chosen for comparison with commercial products (Fig. 11). Most of the imprints, with one exception, confirmed that 5% CHG particles successfully inhibited bacterial growth, reducing microbial counts to single digits per finger.

After 30 min, the effects of ethanolic gel are comparable with no disinfection treatment, given the short-lived effect of alcohol-based products providing only instant protection immediately after the application (Fig. 11, upper array). Alcohol CHG solution showed a better effect than ethanolic gel, although it was less effective than 5% CHG particles. This observation goes along with the fact that CHG has a high affinity to human skin and remains on its surface while ethanol evaporates quickly.

For the test duration of 1 h, the situation was analogous (Fig. 11, bottom array). CHG particles inhibited, to a large extent, bacterial growth, but more emerging colonies were observed. Comparing side-to-side CHG particles and CHG alcohol solution, it is evident that particles provide a similar or higher degree of protection. A subtle effect of lowering microbial counts was observed even for the blank particles (Fig. SI 4). This phenomenon of passive protection can be explained by a steric blockage of the skin surface by micron-sized particles preventing bacteria and other contaminants from attaching and proliferating.

After comparing all results, one volunteer was clearly distinguishable from the rest of the testing group (marked by * in Fig. 11, Figs. SI 2 and SI 3). The nature of activities performed by this subject during the testing period, combined with very dry skin, diminished the effect of CHG particles and commercial disinfections.

## Conclusion

This work describes the preparation and methodology of experimental testing of antiseptic dry powders with a long-lasting protective effect. The micro-groove topology of friction ridges on our fingers provides a relatively large surface area where antiseptic microparticles (chitosan microparticles with incorporated chlorhexidine digluconate) are accommodated and protected against mechanical detachment. Testing with volunteers confirmed the presence of particles even after 2 h of intensive activity. Similar results were observed with a wearable silicon replica of a human finger, allowing for SEM observation of the time-dependent spatial distribution of particles in the fingerprint area. The antiseptic effect of prepared powders was compared with commercial products using the hands of twelve volunteers performing their everyday duties for a specific time. In all instances, the duration and the extent of the protection for 5% and 10% CHG particles were always superior to both tested commercial products, i.e., ethanolic gel and CHG alcohol solution.

Proper hand washing will always be the best action for preventing the spread of infectious diseases. However, when access to running water is limited, or during long-lasting activities (e.g., surgical procedures, rescue operations), dry powder sanitisers activated by human sweat may find their practical use. It is also important to note that alcohol-based sanitisers require a higher application frequency due to their quick evaporation rate and the need for reapplication after every contact with a contaminated surface. However, dry powder formulation offers other advantages, including the absence of alcohol, which may be beneficial for individuals who cannot use alcohol-based sanitisers for cultural or medical reasons (such as dry skin, scrapes, or cracks). Other advantages of dry powder formulations are lower bulk density (i.e., reduced packing size), higher stability of actives in a dry state (i.e., safer storage) or lack of flammable and skin-irritating alcohols. The presented dry powder formulation has myriad possible applications, e.g., incorporated in wound dressings or as the inner coating of powdered gloves, providing an additional layer of protection. In light of these findings, we believe that dry powder sanitisers with long-lasting effects should be considered as an alternative to commonly used liquid-based commercial products.

### Supplementary Information

Supplementary Figures.

## Data Availability

The datasets used and/or analysed during the current study are available from the corresponding author on request.
