# Supplementary material for: Development of spray-dried powder hand sanitiser with prolonged effectivity
Source: Sci Rep. 2024 Feb 28;14:4827. doi: 10.1038/s41598-024-55503-w (PMC10899249; doi:10.1038/s41598-024-55503-w)
Supplement: Supplementary file 1 — Supplementary Figures. [file 41598_2024_55503_MOESM1_ESM.docx]

**Supplementary information**

**Development of spray-dried powder hand sanitiser with prolonged effectivity**

Lucie Večerková^1^, Lucie Mašková^1^, Zdeněk Knejzlík^2^, Ondřej Kašpar^1^, Viola Tokárová^1*^

^1^*Department of Chemical Engineering, University of Chemistry and Technology Prague, Technická 5, Prague 6, 166 28, Czech Republic*

^2^*Institute of Organic Chemistry and Biochemistry of the Czech Academy of Sciences,
Flemingovo náměstí 542/2, Prague 6, 160 00, Czech Republic*

**Corresponding author: tokarovv@vscht.cz*


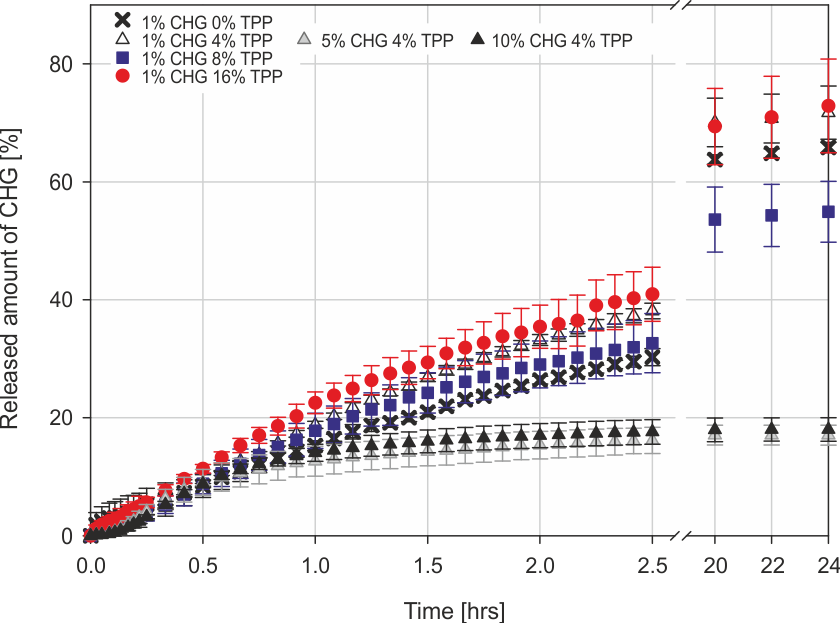


FigureSI 1. Release of CHG (relative amount) from chitosan carriers in PBS.
Note: Encapsulation efficiency was considered for evaluation of the initial CHG amount


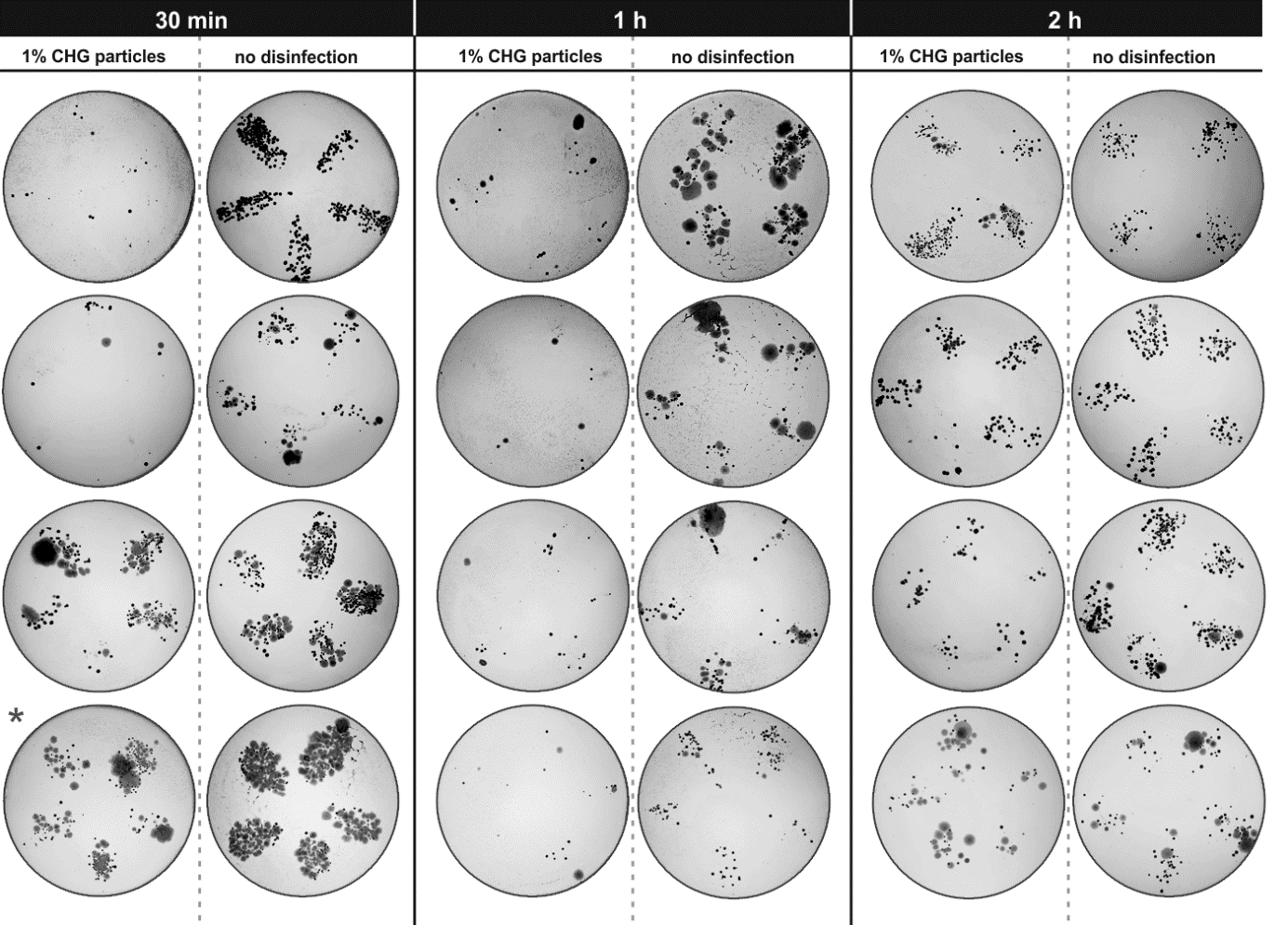


FigureSI 2. The efficacy of 1% CHG particles (test duration 30 min, 1 and 2 hours)





FigureSI 3. The efficacy of 10% CHG particles (test duration 30 min, 1 and 2 hours)


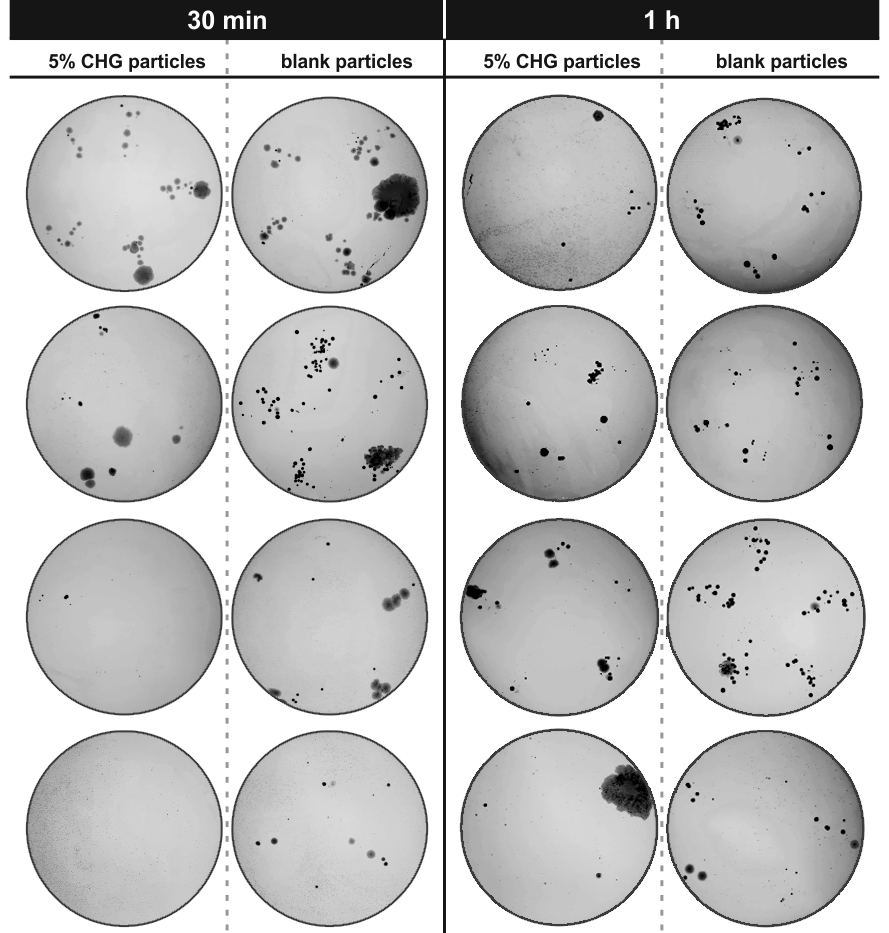


FigureSI 4. The efficacy of 5% CHG particles vs. blank particles (test duration 30 min and 1 hour)
